# A qualitative analysis on the implementation of a nudge intervention to reduce post-surgical opioid prescribing

**DOI:** 10.1186/s12913-025-12651-7

**Published:** 2025-04-08

**Authors:** Meghan C. Martinez, Kathryn Bouskill, Xiaowei Sherry Yan, Allison Kirkegaard, Jason N. Doctor, Katherine E. Watkins

**Affiliations:** 1 Palo Alto Medical Foundation Research Institute & Center for Health Systems Research, Sutter Health, 795 El Camino Real, Ames Building, Palo Alto, CA 94301 USA; 2 RAND Corporation, Santa Monica, CA USA; 3 Center for Health Systems Research, Sutter Health, Walnut Creek, CA USA; 4 Sol Price School of Public Policy, University of Southern California, Los Angeles, CA USA

**Keywords:** Nudges, Behavioral interventions, CFIR, Implementation science, Opioids, Surgeons

## Abstract

**Background:**

Reducing above-guideline opioid prescribing is one approach to reducing the availability of unused opioids. We describe contextual factors affecting the implementation and outcomes of a successful email ‘nudge’ aimed at reducing post-operative opioid prescribing, with the goal of informing future implementation and dissemination efforts.

**Methods:**

Between October 2021-September 2022, we sent email nudges to general, orthopedic, and obstetrics/gynecology surgeons at 19 hospitals in a large integrated healthcare system in California whose patients had post-operative opioid prescriptions that exceeded guideline-recommended quantities. We then interviewed 36 surgeons between September 2022-January 2023 and coded and themed transcripts and implementation process documents from the study. We used the Consolidated Framework for Implementation Research (CFIR) to understand the contextual factors impacting nudge design, implementation, and effectiveness.

**Results:**

Factors across all five CFIR domains were found to be important in understanding the acceptability, feasibility, and sustainability of the intervention. In the Innovation Domain, key factors included the method of nudge delivery, the validity of comparators, and the design and layout of the nudge itself. The interaction between the nudges and existing state regulations (Outer Setting Domain) caused confusion, while the size, structure, and centralization of hospitals (Inner Setting Domain) influenced communication and leadership engagement, underscoring the need for local champions (Individuals Domain). In the Implementation Process Domain, workflow considerations emerged, e.g., the fact that the surgeon performing the procedure was at times not the discharge prescriber, the need for pre-intervention education, and the importance of ensuring surgeons have a clear process to access additional information when questions arise about nudge content.

**Conclusions:**

Contextual factors related to how the nudges were implemented influenced their acceptability among surgeons. Future dissemination efforts of similar interventions to curb opioid overprescribing should take these design considerations into account, including how to account for variations in prescribing workflows, the amount of information provided in the nudge, how information is construed, and how the rest of the hospital system can adjust to encourage guideline-supported opioid prescribing at the point of post-surgical discharge. These types of considerations may also apply to other clinician-directed, nudge-based interventions beyond the subject of opioid prescribing.

**Trial registration:**

Clinicaltrials.gov, Identifier: NCT05070338, Registration Date: October 19, 2021.

**Supplementary Information:**

The online version contains supplementary material available at 10.1186/s12913-025-12651-7.

## Background

Opioids are an important pain management option for patients following surgical procedures; however, research shows that patients are often prescribed more opioids than necessary [1, 2]. Surgeons prescribe 10–20% of all opioids in the United States [3–5], but more than half of the opioids prescribed are never used [6]. Unused prescription opioids are rarely safely discarded and may contribute to chronic opioid use through misuse or diversion [7]. Thus, reducing excessive post-surgical opioid prescribing is likely one important approach to reducing the availability of opioids overall.

In an effort to reduce the quantities of opioids prescribed, a variety of approaches have been implemented across healthcare settings, including strategies aimed at subtly “nudging” clinicians toward making better-informed clinical decisions without ultimately impacting freedom of choice [8–19]. Examples of nudges range from instituting default quantities in the electronic health record (EHR) to providing clinicians with comparison feedback relative to some “appropriate” amount [20]. Many of these behavioral interventions are low-cost and do not directly impact clinical autonomy, and thus hold promise in their acceptability to clinicians and broad scalability across healthcare systems and clinical settings [21]. Yet a recent systematic review of nudge-based strategies to improve clinician practices around evidence-based guidelines found large variation in the size of the nudge effects [21], highlighting the need to consider the target behavior and associated contextual factors that may influence nudges’ impact. In a recent critique of nudges [22], researchers noted that nudges may increase the cognitive burden of clinicians, contribute to decision fatigue, and focus too much on individual behavior at the expense of addressing larger structural issues, such as the push from the pharmaceutical industry to increase prescriptions and, in turn, profits [22, 23]. Understanding how nudge design and the context in which the nudge is implemented may impact outcomes is important for future efforts aimed at disseminating nudges more broadly. While it is important to understand the variability in nudge effectiveness and address the critiques over how nudges may or may not fit within the current United States healthcare system, few studies have systematically considered the process of implementation or the broader context within which they are implemented [21, 24, 25]. Therefore, we undertook a qualitative analysis, one focus of which was contextual and implementation factors of a recent successful clinical trial using nudges aimed at reducing above-guideline post-operative opioid prescribing, with the goal of informing future design and implementation efforts.

We report on the implementation process of two post-surgical nudge interventions implemented in 2021–2022 at Sutter Health, a large integrated healthcare system in California. Surgeons in three surgical specialties (general surgery, orthopedics, and obstetrics/gynecology) were sent monthly email nudges when their patients were prescribed more opioids at hospital discharge than guideline-recommended amounts. Emails contained information on how the prescribed number of opioids compared to either established clinical guidelines or other peer surgeons. We found the nudges were effective in reducing prescribing quantities [26], but interviews with surgeons and implementation process documents highlighted a number of considerations that, if addressed, could possibly further improve the positive impact of these nudges. Therefore, building upon our previous work [27] examining individual-level drivers of change in surgeons’ decision-making heuristics around post-surgical opioid prescribing, we used a common framework for implementation science – the Consolidated Framework for Implementation Research (CFIR) [28] – to elucidate the contextual components impacting the intervention, to explore how factors previously identified interact with the broader organizational contexts, and consider factors important for future deployment in other settings. Our aim was to use this approach to enhance the future efforts to reduce opioid prescribing while allowing for safe and effective pain management and guide the use of future nudge interventions more broadly.

## Methods

### The intervention and setting

**Fig. 1:**
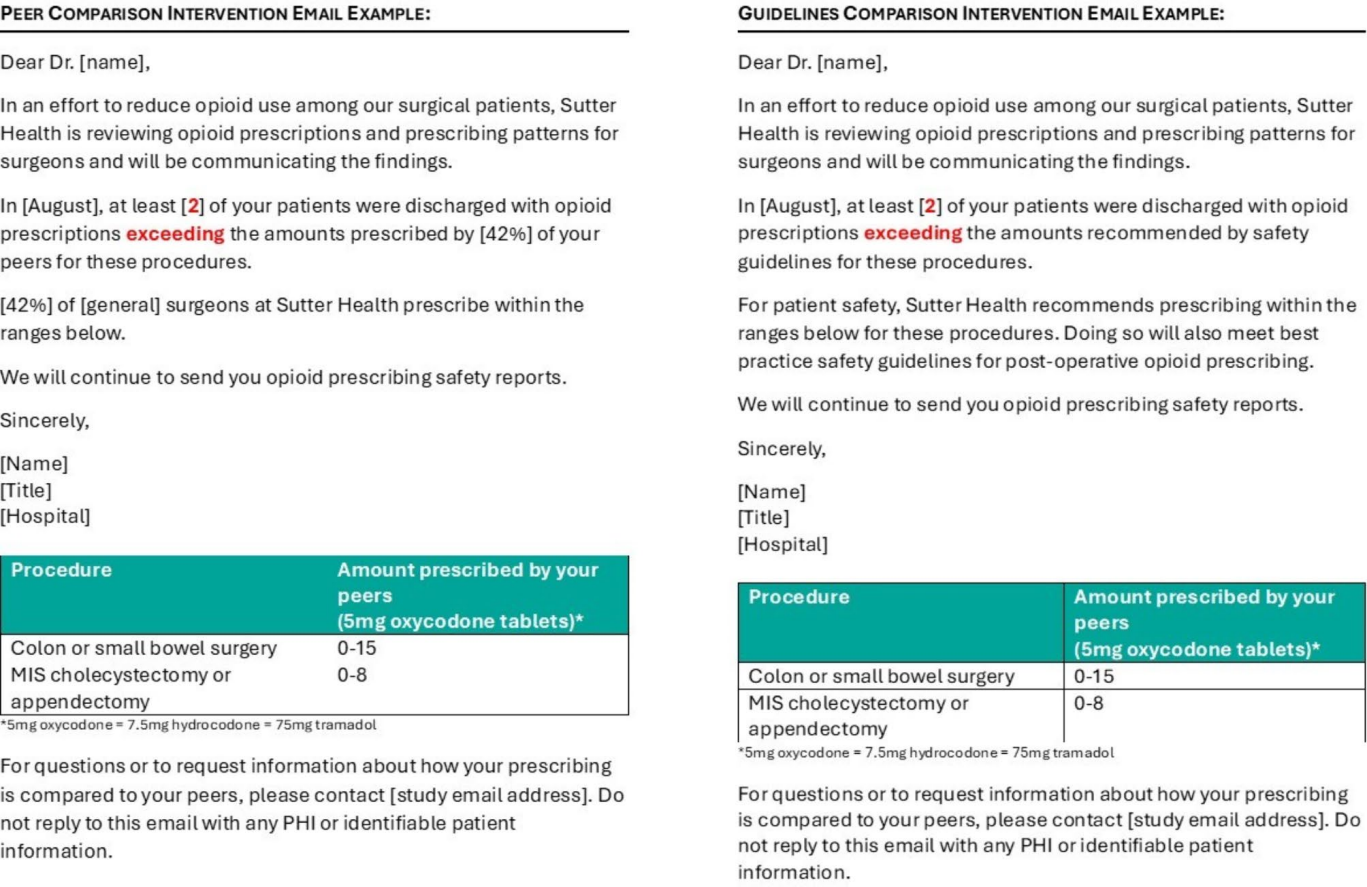
Example nudge language for peer comparison and guideline arms

The full protocol for our intervention has been previously published [29]. The delivery of the two nudge interventions took place between October 2021 and September 2022 at 19 hospitals within the Sutter Health system, a large, multispecialty delivery system covering 23 counties in northern and central California. Surgeons at Sutter Health are either employed by a Sutter Health-affiliated medical group or are independent clinicians with privileges at a Sutter Health hospital. Surgeons within three surgical specialties – general, orthopedics, and obstetrics/gynecology – were cluster-randomized at the department level into one of three groups: (1) a monthly emailed nudge comparing the surgeon’s opioid prescribing behavior relative to that of their peers (“peer comparison nudge”); (2) a monthly emailed nudge comparing the surgeon’s opioid prescribing behavior relative to that of guidelines (“guidelines nudge”); or (3) usual care where no emails were sent (control). Each month a surgeon would receive a nudge only if their patients were prescribed opioid quantities outside of recommended ranges at least two times in the previous month. The ranges came from guidelines developed at the Mayo Clinic [30–32] and are based on patient-reported quantities of actual post-operative opioid consumption after specific surgical procedures. For example, an ACL reconstruction had a range of 0–25 tabs of 5 mg oxycodone (see Supplementary materials). Ranges were presented for 5 mg oxycodone tabs with conversion rates for other opioid types. The research team, made up of researchers, statisticians, economists, and surgeons/physicians in Los Angeles (not part of Sutter Health) and researchers at Sutter Health, designed the study and drafted the nudge language. Prior to implementation, the Los Angeles-based surgeons discussed the email nudge language with colleagues, and the nudges were presented by the Sutter Health research team to Sutter Health leaders for feedback and approval. Attempts were made to pilot test the nudges with a few Sutter Health surgeons, but with limited success despite repeated attempts (with one surgeon simply noting the “wording is fine”). To limit variables impacting outcomes in the larger randomized controlled trial (RCT), the language for the peer and guidelines nudges were made to read as similar as possible. See Fig. 1 for examples of both nudges [29]. The primary outcome was the probability that a discharged patient was prescribed a quantity of opioids above the guideline-recommended amount for that procedure.

Email nudges were sent using an organization-approved email system for bulk distribution (MyEmma). The nudge came from and was signed by a leader specific to each hospital, most frequently the Chief Medical Executive or Chief of Staff of the hospital or the Chair of the specific department. Surgeons received the email nudges at their “preferred” email address rather than their system-issued address because of many surgeons’ looser affiliation with the organization (i.e., surgical privileges) and the concern that not all surgeons regularly checked their organizational email addresses. The nudges were implemented without surgeons’ knowledge to reduce the likelihood of cross-contamination between groups or any pre-implementation modifications to prescribing workflows.

### Qualitative data collection, conceptual framework, and analysis

Data for the analysis came from two primary sources – interviews with surgeons included in the study and implementation process documents. These documents included communication related to obtaining leadership buy-in, feedback during pilot and intervention phases, and research team meeting notes, collected from and recorded by the research team throughout the funding period. We reached out to 245 surgeons via email, with each surgeon receiving at least one invitation to participate. Some surgeons were emailed up to three times in line with our purposive sampling strategy, which aimed to ensure balanced representation across intervention arms and surgical specialties. For the two intervention arms, we recruited surgeons who had received at least one email nudge during the intervention period or who had actively reached out to the research team about the email nudges they had received [27]. Surgeons from the control arm were included to help us understand baseline prescribing workflows, other factors happening at the organization that may have impacted prescribing habits, and whether there was any spill-over from the intervention arms to the control group. A total of 42 surgeons responded and expressed interest in participating in an interview; of these, 6 consented to participate but were ultimately not interviewed due to scheduling conflicts, a lack of response to repeated follow-ups, or our sampling strategy.

We conducted 36 one-time, in-depth telephone interviews with surgeons who responded to our request across each surgical specialty and numerous hospital sites [27]. A semi-structured interview guide was developed for this study by the research team to understand opioid prescribing workflows, reactions to the nudges, and contextual factors impacting nudge effectiveness (see Supplementary materials). Each one-on-one interview lasted between 25 and 60 min, and all were conducted by MM, a female Project Manager employed by Sutter Health with extensive experience in qualitative work and interviewing but with no previous interactions with any surgeon, between September 2022 and January 2023 after the intervention had been stopped. Surgeons were given a $100 gift card as a thank you for their time.

Surgeons consented to participate prior to the start of the interviews and all materials were approved by the Sutter Health Institutional Review Board and RAND’s Human Subjects Protection Committee. At the beginning of the interview, surgeons were given information about the purpose of the interview and assured that their participation was voluntary and confidential. Interviews were audio recorded, transcribed verbatim, and analyzed using Dedoose 9.2.12 [33]. MM also took notes during interviews to supplement audio recordings, when needed. Data analysis was conducted by MM, KB, and AK following the three steps of practical thematic analysis– reading, coding, and creating themes [34]. The codebook was initially created based on the interview guide but was also informed by emerging and novel themes from the data and further contextualized and validated using the implementation process documents. While not formally coded, the implementation process documents were verbally analyzed and discussed by the larger research team to reach consensus on the implementation experience, identifying key patterns, insights, and discrepancies that could either support or refine the themes from the interviews. The details of initial codebook development and coding can be found in a previously published manuscript [27]. In short, MM and KB first developed the codebook based on 3 initial transcripts, then the remaining transcripts were independently coded in parallel by MM, KB, and AK, who continued to update and refine the codebook and met frequently to address discrepancies and reach consensus on coding. Interviewees did not review transcripts and were not invited to provide feedback on findings. MM, KB, and AK determined saturation had been achieved and chose to stop recruitment when the codebook was stable (code saturation), there were no new primary themes emerging from the data, and the authors felt they fully understood the experiences and perspectives of surgeons (meaning saturation) [34, 35].

For this analysis, we focused on codes related to contextual factors that shaped surgeons’ understanding of and reaction to the intervention, existing workflows impacting intervention outcomes, factors related to why a surgeon would or would not like to see the intervention continued, and factors relating to the broader healthcare setting (e.g., department culture). To understand the intervention-related and contextual factors impacting the implementation and outcomes of the nudges, we organized our findings post-hoc into Damschroder et al.’s updated CFIR framework [28] and CFIR Outcomes Addendum [36]. The CFIR framework has been widely used as a framework to evaluate implementation using 5 domains: (1) Innovation; (2) Outer Setting; (3) Inner Setting; (4) Individuals; and (5) Implementation Process. Within each domain are a set of constructs which focus on specific factors to consider for a given domain (e.g., “structural characteristics” within the Inner Setting Domain). The CFIR Outcomes Addendum focuses on both implementation and innovation outcomes using concepts such as “acceptability” and “sustainability” [36]. By revisiting the original codes and applying CFIR post-hoc in a secondary deductive analysis, we were able to reinterpret the data through the lens of the CFIR, and generate new themes that aligned with both emergent and framework-driven findings [37]. See Table 1 for definitions of each of the 5 primary domains and how we operationalized each one for our analysis.

**Table 1:** Definitions for 5 primary CFIR domains and domain operationalization

**Innovation Domain**	
“The ‘thing’ being implemented, e.g., a new clinical treatment, educational program, or city service”*	A nudge to inform surgeons’ behaviors around post-surgical opioid prescribing
**Outer Setting Domain**	
“The setting in which the Inner Setting exists, e.g., hospital system, school district, state. There may be multiple Outer Settings and/or multiple levels within the Outer Setting, e.g., community system, state”*	The larger Sutter Health healthcare system; California’s regulatory policies and requirements; national context (USA)
**Inner Setting Domain**	
“The setting in which the innovation is implemented, e.g., hospital, school, city. There may be multiple Inner Settings and/or multiple levels within the Inner Setting, e.g., unit, classroom, team”*	Three surgical departments (ob/gyn, general, orthopedic) within the 19 Sutter Health hospitals included in the study
**Individuals Domain**	
“The roles and characteristics of individuals”*	Sutter Health leaders and their involvement; surgeons’ professional roles and engagement in the intervention
**Implementation Process Domain**	
“The activities and strategies used to implement the innovation”*	Composition of the research team; shaping of the study design; expanding the innovation’s reach through planning and engagement

*Definitions taken directly from Damschroder et al. [28]

## Results

We interviewed 15 general surgeons, 13 obstetric/gynecologic surgeons, and 8 orthopedic surgeons (*N* = 36): 58.3% were male, almost two-thirds had been in practice between 10 and 29 years, and the majority worked in urban hospital settings, which is consistent with the demographics of our overall study sample (Table 2). In addition, we examined records of our study team’s discussions of factors that informed how we implemented the nudges and included themes in the appropriate CFIR domain. Quotes gathered in interviews are presented below with information on the type of surgeon (general, ob/gyn, or ortho) and intervention arm to which they were randomized (guidelines, peer, or control). Findings are described in detail by CFIR domain with constructs noted, where appropriate, in parentheses (e.g., *Planning*). The results presented below represent common themes expressed by respondents, unless otherwise noted.

**Table 2:** Demographics of study group vs. interviewed surgeons, taken directly from Martinez et al. [27]

	Interview respondents (*N* = 36)	Full study sample (*N* = 640)^a^
***Gender***		
Female	15 (41.7%)	257 (41.7%)
Male	21 (58.3%)	359 (58.3%)
***Urbanicity***		
Rural	16 (44.4%)	322 (50.3%)
Urban	20 (55.6%)	318 (49.7%)
***Years in practice***		
0–9	2 (6.1%)	63 (10.6%)
10–19	13 (39.4%)	161 (27.0%)
20–29	9 (27.3%)	180 (30.2%)
30 or more	9 (27.3%)	192 (32.2%)
***Specialty***		
General	15 (41.7%)	157 (24.5%)
Obstetric/gyn.	13 (36.1%)	297 (46.4%)
Orthopedic	8 (22.2%)	186 (29.1%)
***Study arm***		
Control	11 (30.6%)	218 (34.1%)
Guidelines nudge	15 (41.7%)	216 (33.8%)
Peer comparison nudge	10 (27.8%)	206 (32.2%)

^a^Due to missing data, frequencies do not add to 640 for gender (24 missing) and years in practice (44 missing)

### Innovation Domain

The innovation of this intervention was the delivery of a nudge to inform surgeons’ behaviors around post-surgical opioid prescribing, either by showing adherence to post-surgical opioid prescribing guidelines or comparisons to peer prescribing behaviors.

During initial study design, the study team recognized and discussed a number of possible delivery methods for the feedback-based nudge (*Innovation Design*) – including email, pop-up alerts in the electronic health record (EHR), and paper mail – but determined that paper delivery was too resource-intensive and EHR alerts would have been complicated to program, costly, hard-to-customize, and unwelcomed by both Sutter Health leadership, who did not want to make significant changes to the EHR when success was unproven, and surgeons who had concerns about an EHR-based nudge pop-up interrupting prescribing workflows (“I would be opposed to any further clicks or stops that have to slow us down in [the EHR]” (ortho, guidelines)). While email-based delivery required programmatic support to create data pipelines to track peer comparison data, match surgeons’ prescribing behavior against guidelines, and merge and email out nudges each month, it was flexible and could be modified when changes were needed since the study team created and ran the pipeline (*Innovation Adaptability* and *Relative Advantage*).

The mechanism of the nudge is predicated on the legitimacy of the information presented. In both nudge arms, the evidence base was called into question by some surgeons, warranting further elaboration for subsequent interventions. The guidelines-based intervention arm is premised upon clinicians trusting that the clinical guidelines are established, legitimate, and reflect the best practices for post-discharge opioid prescriptions (*Innovation Evidence Base*). For this study, Sutter Health’s Pharmacy Program chose to endorse those developed by the Mayo Clinic [30–32] because of the breadth and method of development (based in part on actual patient-reported usage). We explored whether surgeons were aware of clinical guidelines chosen for this study and found most surgeons in our interview sample reported being only vaguely aware that guidelines existed (“I think there’s some out there, but I don’t know them” (ortho, guidelines)) and most had not incorporated them into their practice. Additionally, there are multiple groups and/or organizations that have developed guidelines for various surgical procedures [38–40] and, while ranges are relatively similar, there is not one universally accepted set of guidelines. For example, “I think there are guidelines to the multimodal [pain treatments], but I haven’t seen physical guidelines that spell out take this, 500 of this, you know…” (general, control) and an orthopedic surgeon felt there was a need for “better guidelines and I think they need to be more obvious” (ortho, guidelines). Surgeons reported being open to incorporating guidelines into their practice so long as the evidence base behind them was made clear, “it depends on…the depth with which they are researched before they are sent out” (ortho, guidelines) (*Innovation Source*).

Surgeons in the peer comparison arm also questioned the information presented. Several struggled with the term “peers,” feeling it was too ambiguous to be a useful comparator (*Innovation Design*), a difficulty not anticipated by the research team. The research team defined “peers” to include all surgeons within the same surgical specialty at Sutter Health; however, many surgeons were unclear whether peers included others in the same hospital, across Sutter Health, or in the same city, county, state, or country (e.g., “When you say, ‘peers,’ you mean, like, people in my community?” (ortho, peer)). Others considered their peers to cover only the other clinicians in their specific department or group. Similar to questions around the source of the guidelines, ambiguity around the term “peers” also impacted the *Innovation Evidence Base*. Because beyond this question of who comprises “peers,” it was pointed out that “comparing me to my colleagues without knowing how their patients are doing isn’t helpful” (ob/gyn, guidelines) and a comparison to peers might introduce biases, “you know, you have your own personal biases against that peer” (general, peer). On the other hand, it was noted that surgeons tend to be a competitive group by nature and “like to know [how they’re] doing in comparison to everybody else” (general, peer); thus, seeing how others were prescribing allowed them to recognize “[they’re] doing more than [they] need to” (general, peer) and could scale back their prescribing habits. In fact, when asked about continuing the nudges, some surgeons in the guidelines group felt a comparison to peers would be better – “That’s pretty motivating…[to know how] my peers of ob/gyn or OB hospitals [are] doing this work. That would seem interesting” (ob/gyn, guidelines) – while others thought a combination of guideline and peer comparison would be best as long as “peers” was appropriately defined – “Like here’s what the guidelines say, here’s what your group is doing…maybe your group’s out of control and you didn’t know. Maybe you’re the only stickler…” (ob/gyn, guidelines). These suggestions point to potential strategies for future iterations of the intervention, aligning with the *Tailoring Strategies* or *Adapting* subconstructs within the *Implementation Process Domain* discussed below.

Respondents pointed out important considerations for future interventions, again related to the *Innovation Design*. Within the emails themselves, surgeons recommended including a link directly to the guidelines for quick and easy reference. Also, in acknowledging that they “might be guilty of not scrolling” (ortho, peer), there was feedback to put relevant information at the top of the email instead of below the signature block, especially the table of tablet quantities (see Fig. 1):

If you have some important information, put it in the top of the email where the people are going to see it. Don’t expect that they’ve got plenty of time…to be like, ‘I’m going to really dissect this email and make sure I have thoroughly read it’ (ob/gyn, peer).

The table of tablet quantities was created based on 5 mg oxycodone tablets, with a note on conversion to other opioid types (e.g., tramadol); however, it seems that some surgeons may not have looked beyond the oxycodone amounts and discounted the email because of that: “I don’t use oxycodone except in rare circumstances…so the actual level of drug I prescribed is less. I only give Tramadol and usually only 10 pills…so your system is inaccurate” (general, guidelines). Lastly, Sutter Health encouraged the research team to allow for flexibility regarding the signatory of the email; in some cases, the Chief Medical Executive was deemed to be appropriate, while in others, roles closer to the local context (e.g., Department Chair) were preferred. This allowed for the actual email to be adapted appropriately to each hospital department (*Innovation Adaptability*).

### Outer Setting Domain

Many surgeons mentioned factors external to the immediate healthcare setting that they felt had already led them to decrease the quantity of opioids they were regularly prescribing, including the opioid overdose epidemic and advancements in surgical techniques that allowed less painful surgical procedures (*Local Attitudes* and *Local Conditions*). Yet the data our research team collected on baseline opioid prescribing quantities showed that many surgeons were still prescribing 30–40% above guideline ranges in all 3 arms despite these perceived pre-intervention reductions [26].

Surgeons, research team members, and some hospital leaders also noted two other policies that may have already influenced prescribing amounts: California’s Controlled Substance Utilization Review and Evaluation System (CURES) Act, enacted in 2018, and a greater incorporation of multimodal pain protocols, including Enhanced Recovery After Surgery (ERAS) pathways and hospital-based pain services (*Policies & Laws*). CURES is a California state law intended to support monitoring of controlled substances dispensed to patients for the purpose of identifying abuse or diversion. It requires prescribers to consult a prescription drug monitoring database prior to prescribing a controlled substance, but for surgeries it allows for a 5-day supply of post-surgical opioids to be written without consulting the database. Thus, surgeons noted using this 5-day supply calculation for their patients (e.g., one pill every six hours for 5 days = 20 pills) and assumed this was the “appropriate” or “safe” quantity because it was in line with state policy. Surgeons at times assumed CURES was the basis for guideline-recommended quantities noted in the nudge email, and this ultimately created confusion about the ranges in the email nudge. For example, after receiving a nudge, one general surgeon responded about their prescribing quantities, “All are within guidelines– less than 5 days. I don’t see any issues here. I see nothing to change my practice” (general, guidelines), and others mentioned, “the CURES Act thing basically…they make it seem like it’s appropriate to prescribe something for less than five days at post-op” (general, control). With regard to multimodal pain approaches, surgeons repeatedly noted how effective new protocols (*External Pressure*) had already become in reducing patients’ requirements for postoperative opioids: “I actually have really noticed a significant difference since our acute pain service is putting in blocks ahead of surgery…I feel like the opioid use has gone way down…that service has made a big difference in the amount of postoperative pain that people are having” (ob/gyn, control).

### Inner Setting Domain

Sutter Health has a highly experienced data and analytics team, which enabled straightforward and streamlined ramp-up for the critical data analyses for the intervention (*Information Technology Infrastructure*, a subconstruct of the *Structural Characteristics* construct). Despite the existing robust data and analytics team, the intervention still required significant up-front and ongoing programming costs, particularly regarding attribution of the prescription to the operating surgeon.

The size of the Sutter Health organization means hospitals operate largely independently, and there are not always central policies to align with Sutter-wide initiatives or strategies, increasing the complexity for approvals and “buy-in” around the intervention (*Communications*). Communication about the nudge initiative began first with members of Sutter’s executive staff and pharmacy team to garner organizational support for the study and have Sutter Health’s Pharmacy Program select a set of guidelines to endorse. Next, the Sutter Health research team presented the study at various surgical specialty council meetings and clinician leadership teams. Finally, each hospital’s leadership was also approached individually to learn about the project and to obtain approval for implementation at their location. At this stage, one hospital leader felt that the number of initiatives already in place at their hospital was high and did not want an intervention that may impact clinical autonomy and thus opted out (the hospital was included as a control site). The research team recognized that this process was also related to the *Engaging* construct of the *Implementation Process Domain*, discussed later, which focuses on attracting and encouraging participation in the intervention [28].

Departments within Sutter Health also operate relatively independently, with each having developed their own workflows, communication styles, and protocols with fellow care teams (*Relational Connections* and *Learning-Centeredness*, a subconstruct of the *Culture* construct). Across the departments included in our study, we found that these differences sometimes reflected the various sizes of the departments, which ranged widely from 4 surgeons to 91 surgeons. Yet departmental size did not necessarily correlate with any additional coordination or communication about opioid prescribing, as one might expect. Small departments were not necessarily more cohesive in their prescribing habits than larger ones. For example, at one small site it was reported that, “there’s four of us that take call there. We don’t know what each other does…we never really talked about it” (general, control). In contrast, surgeons from a large urban hospital reported having regular discussions on patient opioid use with colleagues even outside of the nudges: “We talk about it because we have a lot of opioid users, so it comes up like, ‘How are we going to deal with this?’” (ob/gyn, guidelines). As a direct result of having received the nudges, some departments reported electing to discuss and develop their own agreement around prescribing quantities, while others chose to ignore the nudges altogether. The research team was not able to establish any overall patterns between departments that ignored the nudges versus those that did not. However, a general surgery department at a suburban hospital with 23 surgeons met as a group because they felt they “were prescribing way too much” and wanted to explore the issue together *(Culture* and *Tension for Change)*. They explained, “we have these multi-disciplinary meetings where topics like this will come up and then we’ll discuss them as a group…to pinpoint where was the issue. A lot of this is sort of automatic–click, click, click–and not really think[ing] about it” (general, peer). That same surgeon also noted that the nudges encouraged them to communicate with their direct reports “that for my post-op patients, this is what I want…and then it becomes a practice amongst my peers in our group, then that’s when we address it during our meetings and then it trickles down from there” (general, peer) (*Culture*). Interestingly, in one case where the consensus amount of opioids was mentioned during an interview, the agreed-upon opioid quantity was higher than the guideline range specified in the nudge: “for anyone having a laparotomy or a cesarean, which is a form of laparotomy, we would prescribe them 15 tablets of 5 mg of oxycodone” (ob/gyn, peer). The guideline-recommended range for a cesarean section is 0–10 tabs of 5 mg oxycodone. In other instances, reasons provided for ignoring the nudges were often related to other CFIR domains, such as concerns about the source of the emails (“these people don’t practice medicine and they’re just kind of telling us what do to” (ob/gyn, guidelines)) (*Innovation Domain*,* Innovation Source)*, the perception that the emails were not appropriately accounting for “what we’re doing” (ortho, guidelines) (*Inner Setting Domain*,* Compatibility)*, relying instead on clinical judgement and shared-decision making with their patients (*Individuals Domain*,* Motivation)*, and the belief that the recommended ranges were too low (“they’re joking if it’s 15 [tabs]” (general, guidelines) (*Innovation Domain*,* Evidence Base)*.

Some surgeons noted how their departments had programmed default quantities in the EHR that did not align with the recommended guideline quantities in the nudge or even CURES requirements (*Compatibility*). This made it more challenging for busy surgeons to adhere to the nudges, particularly because many believed the default amount was the “correct” or recommended quantity, “when you type in Norco [hydrocodone/acetaminophen] for a postpartum C-section patient and the auto-populated tab quantity is 30…watch out for those incongruities” (ob/gyn, guidelines).

### Individuals Domain

As noted in the Innovation Domain above, the research team invested in dialogues with hospital leaders across Sutter to identify and obtain approval for whose name should appear as the sender and at the bottom of the email to show department-level support (*High-Level Leaders*,* Mid-Level Leaders*, and *Opinion Leaders*). Each site was given leeway to choose whom they felt was most appropriate, with some opting for the Chief Medical Executive, Chief of Staff, department chair, or even surgical team members. The research team and upper Sutter Health leadership believed that having a hospital leader’s name associated with the emails would indicate the importance of this intervention and encourage surgeons to actually read the emails (see more about reach and open rates below). Because of the variability in whose name was associated with the nudge email, it is difficult to know how much this impacted nudge effectiveness, though they were equally effective across departments, hospitals, and surgical specialties. While in one case a respondent noted that although the department chair had changed, the nudge had not been updated (i.e., the former chair’s name was still on the email), and other respondents felt that the individual sending/signing the emails was not the appropriate choice (was Chief of Staff from a different surgical sub-specialty), overall, the individual signing the emails was not a frequent point of concern.

But outside of obtaining approval to use one’s name for the email, leaders had limited involvement in the intervention, resulting in a lack of champions at the local level (*Implementation Leads*). The research team identified a few reasons for this. First, interactions were primarily virtual. Because of the large footprint of the organization, the number of leaders across all levels of the healthcare system that needed to provide approval, and the timeline imposed by grant funding, it was infeasible to meet all necessary leaders in person. Presentations over remote video conferencing platforms or email communication ultimately required less attention and engagement. Second, as an RCT, the research team wanted to retain control over the implementation for consistency of methodology and delivery. On the one hand, this may have helped garner buy-in for the intervention as no time commitment was required from busy hospital leaders (who often also had their own medical practice), but it also meant that when departmental leaders (*Opinion Leaders*) were approached about the email nudges from surgeons in their departments, their responses were less than supportive: “…it’s interesting because it came up with the current chief of our department and she’s like, ‘I don’t know what you’re talking about’…she had no idea these things were coming out…because it comes with her name on it” (general, peer) and a Chief of Staff telling a surgeon “I got to send those out to everybody” (ortho, guidelines). Third, the lack of involvement from leaders also limited the research team’s knowledge and awareness of local differences, for example with pre-programmed EHR order sets that did not match guideline ranges, which come up during interviews.

Another consideration is that the nudges were not designed to speak directly to the constraints of each surgical specialty (*Innovation Recipients*). However, all respondents spoke to how their specific surgical specialty influenced their opioid prescribing behaviors, especially as it related to the need to weigh the impact of opioids on post-surgery recovery. For example, orthopedic surgeons felt that higher amounts of opioids may be warranted to allow patients almost immediate use and movement of joints, while ob/gyn surgeons had to consider a new mother’s pain against opioid impacts on breastfeeding and the negative side effects of constipation after C-section. General surgeons who specialized in breast surgery were unique in that “most fellowship-trained breast surgeons do not routinely prescribe opioids for patients” (general, guidelines). The research team chose to not include any tailoring of the nudge text to ensure uniformity, but as surgeons pointed out in interviews, doing so may have been impactful for addressing some of their specialty-specific concerns.

Local conditions (a component of the *Outer Setting Domain*), for example the lack of access to refills should a patient require more medication, impacted surgeons’ view of their role as a provider and became an ethical concern for surgeons, a contextual factor not foreseen by the research team (*Motivation*, part of the *Characteristics subdomain*). Sutter Health serves a geographically diverse patient population covering 23 counties all with rural, urban, and suburban communities. Thus, patients may need to travel long distances for surgery at a Sutter Health hospital, and they may live in areas with no or few nearby pharmacies or have access only to pharmacies with limited hours. Even in urban and suburban settings, there was a growing concern about pharmacy closures and consolidations. This influenced some surgeons’ decisions not to reduce their prescribing quantities in an effort to “do no harm” to their patients and was simply stated, “I don’t want them to suffer” (general, peer). Others questioned the appropriate balance in opioid prescribing, “So, I guess we have to ask ourselves the question, are we trying to be patient centric on the not under-prescribing side? Or, I’m not sure what the centric is on the not prescribing too many narcotics. And so, we’re willing to take a whole bunch of extra phone calls and making patients suffer in pain. So, I’m just trying to figure out what the goal of this thing is” (ob/gyn, guidelines). In essence, the nudge text did not specifically address concerns around ethical considerations that surgeons raised, but as we learned, this could also have impacted whether a surgeon changed their prescribing behavior or not.

### Implementation Process Domain

As noted, this study was implemented as an RCT and the study team deliberately decided not to inform the majority of providers about the study to limit any Hawthorne effect, where individuals change their behavior simply because they know they are being observed. However, surgeons repeatedly noted a lack of awareness of the intervention and how this impacted their receptivity to the emailed nudges (*Innovation Recipients*, a subconstruct of the *Engaging construct*). Some noted that they felt as though they were being chastised for something they did not know they were doing wrong: “When I deal with my children, I do not lecture them for doing something wrong that they didn’t know was wrong until after I’ve told them about it and we’ve talked about it. The email came off like a lecture without the previous accompanied (sic.) information” (general, guidelines). Respondents wanted additional information around the guidelines, how they were created, and what evidence base there was for them. Specifically, one surgeon wanted to know “what percent of [their] patients were *not* going to have their pain adequately controlled” if they followed the guidelines (ob/gyn, guidelines) (*Assessing Need)*. Surgeons also asked for additional information on the specific patients and procedures that were being called out in the nudges, often because they wanted to investigate the specific patients themselves, see what they prescribed, and decide whether the prescription was appropriate (*Assessing Needs* and *Assessing Context)*. Additionally, they felt pre-intervention education would have been appropriate to notify surgeons about this new program and set expectations around receiving feedback to reduce confusion. The research team prepared standard responses to anticipated questions in advance of the intervention and included in the nudge emails an email address to which questions could be directed, but fewer than 50 of the 640 surgeons reached by the intervention chose to contact the study team.

The research team, which did not include any surgeons practicing at Sutter, elected to send nudges to the surgeon performing the surgical procedure as recorded in the EHR (*Innovation Recipients*). This choice was made for two reasons: (1) to reduce the likelihood of cross-contamination among the intervention arms (if other staff writing prescriptions support multiple surgeons), and (2) because the research team, including four surgeons outside of Sutter, believed the performing surgeon was the individual ultimately responsible for the patient’s care. However, interviews revealed that attributing prescriptions in this way at times negatively impacted the acceptability of the intervention, particularly for ob/gyn and other shift-based surgeons who were on-call for a weekend or evening but were not working when the patient was discharged. Some surgeons noted having reduced their prescription quantities but still received nudges, eventually leading them to ignore the emails because they “figured…that it was because other people were discharging my patients or something like that” (general, peer).

We examined factors that might have impacted the reach of the email nudge, in particular the open rate of the emails (*Implementation*, a subconstruct of the *Reflecting & Evaluating construct*). In pre-implementation discussions with surgeons and leadership, both noted the concerns about email fatigue while recognizing the relative ease of email delivery versus other options. Leadership recommended we use a surgeon’s “preferred” email address, as opposed to simply using the system-issued email address, because many surgeons have looser affiliations with the healthcare system and may not regularly check their system address. As part of implementation, we tracked the open rate of emails – over the year of the emailed nudges, the open rate for the peer group ranged from 39 − 56% and for the guideline group from 36 − 68%.

### Implementation and innovation outcomes from the CFIR outcomes addendum

The CFIR Outcomes Addendum focuses on anticipated implementation outcomes, actual implementation outcomes, and innovation outcomes, and includes key components of acceptability and sustainability [36].

We measured the effectiveness of the nudges, an Innovation Outcome, to evaluate their impact on opioid prescribing practices. Over the 12-month cluster-RCT, both the peer comparison and the guidelines arms showed significant reductions in guideline-discordant opioid prescriptions. These reductions continued to be maintained 12 months after the last nudge was emailed.

In interviews, we investigated acceptability of the intervention, an Implementation Outcome, by asking surgeons their opinions on whether the emails should continue to be sent out and potential considerations that could improve acceptability in future nudge efforts. Several surgeons felt that the nudge was valuable as a reminder and that receiving it via email was not burdensome, noting, “They’re welcome to send them. It’s fine. It’s not very obtrusive at all.” For surgeons in favor of continuing to receive the email, they noted the need to directly attribute the prescription back to the prescribing clinician, which may or may not have been the surgeon. Others called for improvements to the email by adding contextual data to help understand the magnitude of overprescribing (i.e., if the overprescription was a rare occurrence or a continuous trend) and patient safety-related metrics (e.g., did patients use all the medication? Did they have any left? Did they require a refill? ). The importance of limiting leftover pills was mentioned as a consideration for some respondents, “when I ask patients about it they’re like, ‘Oh, I’m not using [them].’ And I don’t want people to have those pills just laying around their house. Because they’ll either take them when they’re not supposed to or someone will steal them” (ob/gyn, control). Lastly, surgeons called out the need to have better feedback mechanisms if the intervention were to be continued so that they could more directly ask questions and share opinions. Table 3 maps the relevant contextual factors influencing nudge implementation to key recommendations uncovered from interviews, process documents, and research team experiences.

**Table 3:** Contextual factors influencing nudge implementation and key recommendations, by CFIR domain

Contextual factors	Recommendations based on interviews, process documents, and research team experiences
**Innovation Domain**	
❖ Design of the method of intervention delivery (*Innovation Design; Innovation Adaptability; Innovation Relative Advantage)*	❖ Evaluate whether email would afford the same advantages (e.g., it involved fewer programming requirements than EHR nudge, could be more easily changed/updated, and required no additional clicks or stops in the EHR)
❖ Evaluation the validity of the comparator(s) *(Innovation Design; Innovation Evidence Base)*	❖ Consider using both guidelines and peer comparison; clearly define “peers” for recipients; provide information on source and evidence base of the guidelines
❖ Adjustment of the structure and layout of nudges *(Innovation Design)*	❖ Put relevant information (e.g., recommended prescribing ranges) toward top of email; include links to guideline evidence base
**Outer Setting Domain**	
❖ Determine external *policies and laws* impacting opioid prescribing and their specific requirements	❖ Clarify any discrepancies between legal prescribing requirements and nudge-recommended prescribing quantities
**Inner Setting Domain**	
❖ Assessment of *information technology infrastructure* (a subconstruct of *Structural Characteristics*)	❖ Ensure data and analytics capabilities. Available data and analytics team allowed for successful implementation, but still required up-front and ongoing programming support and personnel time and involvement
❖ Evaluation of departmental size and culture (*Relational Connections; Communications; Learning-Centeredness*, a subconstruct of *Culture )*	❖ Discern whether departmental size could change the effect. In this case, departmental size did not correlate with discussions or coordination on opioid prescribing
❖ Determination of centralization within the Inner Setting (*Relational Connections; Communications*)	❖ Effort required to engage leaders at each site over large geographic area; alignment of intervention with site-specific workflows (e.g., pre-programmed quantities in EHR)
**Individuals Domain**	
❖ Engage local champions (*High-level Leaders; Mid-level Leaders; Opinion Leaders; Implementation Leads*)	❖ Investigate relevance to nudge recipients of who signs/sends emails; involve local champions to lend support to intervention at department level; use local champions to identify local/department-level variations to consider
❖ Respond to surgeons’ professional role (*Motivation*, part of the *Characteristics subdomain*)	❖ Recognize the surgeon’s ethical commitment to their patients and desire to “do no harm”
**Implementation Process Domain**	
❖ Receipt and review of nudges by recipients (*Tailoring Strategies*)	❖ Use “preferred” email address when clinicians may have looser affiliation with the healthcare setting; use a system to track open rates of emails; consider levels of email fatigue
❖*Assess[ment of] needs and context* of recipients during pre-implementation phase	❖ Recognize and address recipient’s priorities and preferences for pre-intervention education; distribute guideline ranges; provide avenue for surgeons to access additional information (i.e., when questioning patient or procedure information in nudge)
❖*Adapt[ation]* the nudge to prescribing workflows	❖ Attribute prescribing to individual writing prescriptions/discharging patient

Other considerations from the research team affecting long-term sustainability included operational costs, including a dedicated team for producing and sending nudges each month, continuous maintenance for personnel changes, constant quality checks and availability for fielding questions and feedback, and limited buy-in affecting appropriate devotion of resources.

## Discussion

In this paper, we used a systematic approach based on CFIR to identify factors affecting implementation and outcomes of two email-based nudges aimed at reducing post-surgical opioid prescribing. Our findings underscore the complexity of integrating nudges into clinical workflows, with challenges identified across all CFIR domains, and we present considerations to enhance the feasibility, acceptability, and sustainability of future nudge interventions. Additionally, our findings emphasize the complex interplay of CFIR domains that impact acceptability of such interventions.

One key aspect we explored was the mode of delivery: a monthly email nudge sent to the prescribing surgeon. This approach seemed advantageous in its simplicity, seamless integration into existing workflows, preservation of autonomy when making prescribing decisions, and ease of modification. Nevertheless, we found it still required substantial back-end personnel and IT resources to calculate when and to whom the nudge would be sent, as well as any relevant surgeon-specific information to include. The ultimate success of the nudges in reducing post-surgical opioid prescribing amounts relied heavily on how well they complemented and integrated with the context in which they were implemented, and we found tradeoffs due to the inability to control for all possible variations across different domains. For example, we found that the EHR system created a challenge in aligning default opioid prescription quantities with the guidelines provided in the nudges. Surgeons expressed confusion when the EHR’s amounts exceeded nudge quantities, with at least one surgeon who believed the discordance between guidelines and defaults diminished intervention effectiveness. Modifications to the EHR, such as pre-programmed default quantities, have been shown to increase rates of guideline-concordant prescribing and reduce keystrokes for clinicians [41–43], particularly in settings where concordant prescribing was low at baseline [42]. Addressing this issue in future use of the nudge by adjusting EHR defaults to align with guideline-recommended quantities would further enhance the effectiveness of the intervention.

In the Outer Setting Domain, we found that the California CURES Act had already decreased opioid prescribing quantities (*Policies & Laws)* [44]. However, some surgeons expressed confusion about how exceptions for post-surgical prescriptions under the CURES Act aligned with the guideline-recommended quantities in the nudges. Surgeons were often surprised to find that their prescribing was “excessive,” questioning whether further reductions were necessary. The overlap between policy and nudge highlights the need for clearer alignment between policies and external factors within the Outer Setting domain and intervention guidelines. Provided that nudges and policies are aligned, a combination of policy/law (e.g., CURES) and nudges would be more effective in bolstering and sustaining behavior change.

In the Innovation Domain, *Innovation Design* and *Innovation Evidence Base* emerged as the most salient constructs and highlighted how the success of the intervention was impacted by the message and language fitting with the specific practice environment. Importantly, some surgeons did not believe the nudges sufficiently addressed their concerns about patient care, especially that reducing the amount of opioid prescribed would not increase pain and suffering. These concerns, rooted in the Individuals Domain and the surgeon’s professional role, reflected the ethical motivation of surgeons to “do no harm” to their patients (*Motivation*, part of the *Characteristics subdomain)*. Therefore, the research team believed future nudges may benefit in educating and directing attention to the high number of excess opioid pills per prescription within current prescribing practices, a component absent from our study. Such an approach would address surgeons’ ethical concerns about adequate patient pain control and patient suffering. Furthermore, measuring patient satisfaction with their post-surgical pain and relaying this information back to surgeons could be another important component for future interventions, although this was prohibitively resource-intensive in our study.

The Inner Setting Domain revealed the complexity of implementing a one-size-fits-all nudge intervention across different hospital departments, even within a single healthcare system. Consistent with other research showing departmental-level factors, such as the size and culture, impact the acceptability of change initiatives [45, 46], we found that some departments were more cohesive and receptive to behavioral change, others were less so. These differences were not always in the expected direction (e.g., small departments more cohesive), making it difficult to understanding the *Relational Connections* and *Compatibility*, even for research team members embedded in the system. Furthermore, reasons provided for ignoring the nudges were linked to numerous CFIR domains, such as questions about the email sources (related to the *Innovation Domain*,* Innovation Source)*, the autonomy of clinical practice (related to the *Innovation Domain*,* Innovation Source* and the *Individuals Domain*,* Motivation*), and doubts about the appropriateness of the recommended ranges (related to the *Innovation Domain*,* Evidence Base).* The diversity of departments also required a tailored approach to gaining buy-in from various stakeholders and emphasized the interplay between the Inner Setting Domain and Individuals Domain. Specifically, *Communications* at the department-level within the Inner Setting Domain, including engaging local champions (*High-level Leaders*,* Mid-level Leaders*, and* Opinion Leaders)* and allowing each department to choose the most appropriate person to sign the email--both of which were factors housed within the Individuals Domain--seemed to be key in overcoming departmental resistance.

The Implementation Process Domain revealed that surgeon feedback was essential in refining the nudges to better integrate them into prescribing workflows and identifying relevant contextual features that might have otherwise been underappreciated by the research team (*Innovation Recipients*, a subconstruct of the *Engaging construct*). Particularly in efforts to decrease the amount of opioids prescribed, these “experience[s] of the provider” have often been overlooked [41]. Surgeons reported that they often ignored the nudges because they were sent to the operating surgeon rather than the prescribing clinician. In future implementation efforts, sending the nudge to the clinician associated with the prescription could lessen programmatic efforts as fewer data elements would need to be linked from the EHR while simultaneously increasing surgeons’ acceptability of the nudges. Additionally, offering surgeons the option to choose their “preferred” email address for receiving nudges could enhance the effectiveness as it may ensure the information presented is actually read. Although open rates seemed reasonable based on the research team’s prior experiences, future efforts may want to consider periodically changing the subject line to keep the surgeons engaged *(Engaging)* and monitoring open rates to determine what is considered acceptable rate for a given organization. Surgeons also recommended more pre-implementation education about the evidence base behind the guidelines and the rationale behind reducing opioid prescribing to within recommended ranges (*Assessing Context*). Future nudge efforts should consider better preparing surgeons for change, adding additional details, including on the patients or procedures called-out in the nudge, and providing an avenue for accessing additional information when questions arise to further improve the intervention’s acceptance.

While surgeons overall were open to continuing to receive the nudges, they had numerous recommendations that would further improve acceptability. An important aspect of the intervention’s design was the formatting of the nudge. Several surgeons recommended changes to make it easier for clinicians to quickly find and understand relevant information, such as placing recommended prescribing ranges at the top of the email and providing an active link to the guidelines. Surgeons admitted to being “guilty of not scrolling,” therefore missing important information and undermining the effectiveness of the nudge. Streamlining the layout to make the guidance more prominent aligns with the broader findings from the Implementation Process Domain, where more systematic user experience/user interface research from clinicians during the pre-implementation phase would have likely elucidated the importance of language and structure to better align with clinical workflows.

These findings suggest that while nudges alone can drive initial behavior change, the long-term sustainability of such change remains uncertain. To achieve sustained changes, broader system-level support–like education, alignment of policies, and organizational backing—seem to be critical to reinforcing the nudges [22]. This study also provides insights for the emerging field of *de-*implementation science [47], which focuses on reducing or eliminating ineffective or harmful practices. In that context, this study explored the implementation of an intervention designed to de-implement an unwanted behavior. *De*-implementation science addresses the complex challenges around reducing practices like medication over-prescribing, where clinician resistance often stems from concerns around harms to patients, adequate pain control, and ethical concerns regarding their role as independent decision-makers to support patient well-being [48]. Future research would benefit from a roll-out implementation optimization approach [49, 50] or similar strategy using real-time surgeon feedback to adapt and sustain nudge interventions within the context of de-implementation, with an emphasis on continuous feedback, ongoing clinician education, policy alignment, and institutional investment [51]. Our findings made clear that the provision of information was ultimately more critical than the nudge per se. Educational efforts that go beyond the nudge, such as departmental meetings, leader champions, and institutional culture shifts, are necessary to facilitate sustained change. In the end, providing surgeons with accurate, actionable information was key to changing behavior, and while nudges can serve this function, they must be paired with broader efforts to embed sustained behavior change.

###  Limitations

This study is not without limitations. The project was implemented at a single healthcare organization in northern California, which may not be representative of all organizations. However, the footprint of the organization is large, with 19 unique hospitals across both rural and urban areas. We did not do systematic interviews with Sutter or hospital leadership and thus have a limited understanding about their perception of implementation. The research team did not include any Sutter Health clinicians/surgeons who may have been able to help further refine the nudge prior to implementation (e.g., nudge language and structure). The surgeons who agreed to participate in interviews may not be representative of all surgeons receiving a nudge, and recruitment of surgeons proved to be challenging, particularly among orthopedic surgeons. Social desirability bias is possible, as surgeons may have underreported negative perceptions to not seem reluctant to engage in efforts to curb the opioid overdose epidemic. This study also does not provide for a direct comparison of nudge-based interventions to policy-based interventions (i.e., top-down interventions), which has been noted in the literature as an important gap in understanding [21]. Nevertheless, we did ask surgeons about factors within the Inner and Outer Setting Domains that may have impacted prescribing habits separate from the intervention and reported those findings. We did not systematically assess whether there were other nudges happening within the system and the potential for decision fatigue [23, 24].

## Conclusions

Interventions designed to collect and share information with clinicians about opioid prescribing behaviors at the point of post-surgical discharge are one proven method of curbing opioid overprescribing. However, the successful implementation of these interventions requires close attention to the clinical setting, workflows, departmental culture, leadership involvement, and the design of the intervention itself in terms of content and delivery. For nudge-based interventions like the one explored here, future interventions should carefully weigh the tradeoffs regarding programming efforts, including addressing surgeon concerns around the language, content, and structure of the email, as well as attribution of the prescription back to the prescribing clinician. Ensuring alignment between the nudge and other hospital practices, like default EHR settings, and harmonizing with existing policies and laws within the Outer Setting Domain, will enhance the intervention’s effectiveness. Additionally, the pre-implementation efforts should focus on educating clinicians, addressing cultural acceptability, and fostering leadership involvement to support the broader success and sustainability of nudge strategies aimed at changing clinician behaviors.

## Supplementary Information

Supplementary Material 1.

Supplementary Material 2.

## Data Availability

Data are available upon reasonable request. We are able to provide (1) deidentified demographics (without site), but no contact information; (2) interview protocols; and (3) the qualitative analysis codebook. Reuse is permitted for similar qualitative studies with appropriate citation. Data are available from Meghan Martinez (meghan.martinez@sutterhealth.org).

## Abbreviations

CFIR: Consolidated Framework for Implementation Research
EHR: Electronic Health Record
RCT: Randomized controlled trial
CURES: California’s Controlled Substance Utilization Review and Evaluation System Act
ERAS: Enhanced Recovery After Surgery
ER: Emergency Room
CEO: Chief Executive Officer
CMO: Chief Medical Officer
