# Supplementary material for: A qualitative analysis on the implementation of a nudge intervention to reduce post-surgical opioid prescribing
Source: BMC Health Serv Res. 2025 Apr 8;25:512. doi: 10.1186/s12913-025-12651-7 (PMC11977946; doi:10.1186/s12913-025-12651-7)
Supplement: Supplementary file 1 — Supplementary Material 1. [file 12913_2025_12651_MOESM1_ESM.docx]

**POST-OP:** POST-Operative nudges to reduce Opioid Prescribing

Post-Intervention Interview Protocol

Do I have your consent to record this interview? [Y/N]

1. **Introduction**
2. We would like to begin by learning about your background (e.g., training, clinical or administrative focus).
3. How long have you been at [site]?
4. How often do you perform [procedure(s)]?
5. **Prescribing Workflows**
6. Please walk us through the process of prescribing opioid pain medication for an average patient receiving [list of possible procedures] at the time of discharge.
   1. When are discussions on pain medication type and amount initiated (pre-op, post-op, etc.)?
      1. To what degree does this differ across your patient panel?
      2. How do you factor in other prescriptions a patient may be taking (e.g., if a patient is taking benzodiazepines)?
      3. Do you have specific order sets?
      4. How often do you change a prescription based on patient preferences?
      5. What do you think is unique to prescribing pain medication in your specialty?
      6. What do you think is unique to prescribing pain medication in this facility?
      7. What do you think providers could do to ensure that patients receive the appropriate amount of prescription pain medication?
7. Are you aware of any clinical guidelines for opioid prescriptions at discharge following [procedure]? [could mention CURES, et cetera]
   1. Do you discuss opioid prescribing with your colleagues at [facility]?
   2. Do you discuss opioid prescribing with your professional groups?
8. How do you in general react to practice guideline alerts?
9. How has COVID changed opioid prescribing, if at all?
   1. Has there been anything that has prompted you to change opioid prescriptions over the last year?
10. In general, how much does the opioid epidemic factor into your thoughts on prescribing opioids to patients for [procedure(s)]?

[For control arm interviewees, ask if there is anything else regarding opioid prescribing that was not discussed.]

**[For prescribers who received the intervention]**

1. Do you recall receiving an email with how the prescriptions for opioids you compared with [clinical guidelines] [how your colleagues prescribe opioids]?
   1. If so, how do you remember reacting to this email?
      1. What did you like about it? What did you not like about it?
   2. Was the information presented new to you?
   3. [If applicable] Do you recall reading through the guidelines table at the bottom of the email?
      1. If so, what did you think of them?
      2. How appropriate/realistic did the quantities in the table at the bottom of the email seem to you?
      3. How did you feel about after realizing that your prescriptions were non-compliant with guidelines?
   4. [If applicable] Do you recall seeing how your prescribing compared with that of your colleagues?
      1. How appropriate/realistic did the percentages seem to you? How appropriate/realistic did the quantities in the table at the bottom of the email seem to you?
      2. How did you feel after realizing that your prescriptions were not in line with the amount your colleagues were prescribing?
   5. Did you discuss the email with your colleagues? If so, what were their reactions?
2. Did the email prompt you at all to reconsider the amount of opioids you prescribed for patients at discharge following [procedure]?
   1. Did you start to track/think about the amount you were prescribing differently after receiving this email?
   2. [If applicable] Were there other factors that influenced you to change your prescriptions?
   3. [If applicable] Are there other factors that influenced you to continue to prescribe similar amounts?
3. Would you want to keep receiving similar information in the future?
   1. If so, what might be the best way for you to receive this information?
4. **Conclusion**

1. Is there anything that we have not discussed today regarding the process of prescribing pain medication for your patients that you think would be important to understand?

**[Thanks and conclude interview]**
