# Supplementary material for: A qualitative analysis on the implementation of a nudge intervention to reduce post-surgical opioid prescribing
Source: BMC Health Serv Res. 2025 Apr 8;25:512. doi: 10.1186/s12913-025-12651-7 (PMC11977946; doi:10.1186/s12913-025-12651-7)
Supplement: Supplementary file 2 — Supplementary Material 2. [file 12913_2025_12651_MOESM2_ESM.docx]

Opioid Clinical Guidelines for Surgical Procedures

| **Procedure** | **5mg oxycodone tabs** |
| --- | --- |
| ACL reconstruction | 0–25 |
| Acute fracture management | 0–15 |
| Ankle arthrodesis | 0–40 |
| Ankle fracture ORIF | 0–40 |
| Bronchoscopy or upper endoscopy | 0 |
| C-section | 0–10 |
| Carotid endarterectomy | 0 |
| Carpal tunnel release | 0–15 |
| Colon or small bowel surgery | 0–15 |
| Distal radial ORIF | 0–40 |
| Endoscopy | 0 |
| Femoral-neck fracture ORIF | 0–25 |
| Gynecologic laparotomy (hysterectomy, omentectomy, lymphadenectomy, etc.) | 0–5 |
| Hysteroscopy | 0 |
| Knee arthroscopy | 0–25 |
| Lumbar laminectomy or laminotomy with arthrodesis | 0–50 |
| Lumbar laminectomy or laminotomy without arthrodesis | 0–40 |
| Major spine surgery | 0–50 |
| Mastectomy with subcutaneous reconstruction | 0–15 |
| Mastectomy with submuscular reconstruction | 0–30 |
| Minimally invasive gynecologic surgery (laparoscopic or robotic) | 0–5 |
| Minor spine surgery | 0–40 |
| MIS abdominal solid organ resection | 0–15 |
| MIS bariatric, benign foregut, or adrenal surgery | 0–8 |
| MIS cholecystectomy or appendectomy | 0–8 |
| MIS inguinal hernia repair | 0–8 |
| MTP arthrodesis | 0–25 |
| Muscle biopsy or excisional biopsy | 0 |
| Open inguinal hernia repair | 0–8 |
| Open major abdominal resection | 0–30 |
| Percutaneous endovascular or vascular access procedure | 0 |
| Shoulder arthroscopy | 0–40 |
| Simple mastectomy | 0–10 |
| Thoracotomy (pulmonary, pleural, or chest wall) | 0–50 |
| Thumb basal joint reconstruction | 0–25 |
| Thyroid/parathyroid surgery, mediastinoscopy, or POEM | 0–5 |
| Total hip arthroplasty | 0–50 |
| Total knee arthroplasty | 0–50 |
| Total shoulder arthroplasty | 0–50 |
| VATS procedure (pulmonary or mediastinal) | 0–20 |
| Wide local excision or lumpectomy | 0–5 |
